# From trauma capacity to neurotrauma capacity: A scoping review of existing assessment frameworks

**DOI:** 10.1016/j.bas.2026.107474

**Published:** 2026-10-12

**Authors:** Nicolò Marchesini, Dina Belhaj Youssef, Ellianne Dos Santos Rubio, Edoardo Picetti, Tariq Khan, Corrado Iaccarino, Andrès M. Rubiano

**Affiliations:** a Department of Neurosurgery, Azienda Ospedaliera Universitaria Integrata di Verona, Verona, Italy; b Leiden University Medical Center, Leiden, the Netherlands; c Department of Neurosciences, Biomedicine and Movement Sciences, Section of Neurosurgery, University of Verona, Italy; d Department of Neurosurgery, Willemstad, Curaçao; e Department of Anesthesia and Intensive Care, Parma University Hospital, Parma, Italy; f Northwest General Hospital and Research Centre, Peshawar, Pakistan; g School of Neurosurgery, Department of Biomedical, Metabolic and Neural Sciences, University of Modena and Reggio Emilia, Modena, Italy; h Neurosurgery Division, University Hospital of Modena, Modena, Italy; i Neurosurgery Unit, AUSL RE IRCCS, Reggio Emilia, Italy; j Meditech Foundation, Cali, Colombia; k Department of Neurosurgery, Universidad del Valle, Cali, Colombia; l Neuroscience Institute, Universidad El Bosque, Bogota, Colombia

**Keywords:** Neurotrauma, Capacity assessment, Health systems, Global surgery, Scoping review, Developing countries

## Abstract

**Introduction:**

Neurotrauma, comprising traumatic brain injury (TBI) and traumatic spinal cord injury (tSCI), remains a major global health issue, with low- and middle-income countries (LMICs) bearing a disproportionate burden. Effective neurotrauma care requires timely and coordinated access to prehospital care, imaging, surgery, neurocritical care, and rehabilitation, yet current assessment frameworks often fail to capture these interdependent requirements.

**Research question:**

What instruments are currently available to evaluate trauma and neurosurgical capacity across diverse global settings, and to what extent do these tools capture indicators relevant to neurotrauma care?

**Material and methods:**

We conducted a scoping review following JBI methodology and PRISMA-ScR guidelines. We searched PubMed/MEDLINE, Embase, Web of Science, and Global Health from inception to March 2026, supplemented by grey literature. We included studies describing instruments assessing system or facility-level capacity for trauma or neurosurgical care. Data extraction and domain mapping were performed independently by two reviewers.

**Results:**

Twenty-six documents were included, describing 19 distinct instruments. Instruments were categorized as surgical capacity (n = 4), trauma capacity (n = 4), neurosurgical/neurocritical care capacity (n = 4), and quality indicator sets (n = 7). Emergency and imaging readiness was the most frequently covered domain (89%), followed by quality and audit systems (68%), operative capacity (53%), and neurocritical care (47%). Prehospital referral systems and workforce/training were the least covered domains (37% each). Only two instruments covered all six predefined neurotrauma domains.

**Discussion and conclusion:**

Existing tools assess important components of trauma and neurosurgical capacity but remain heterogeneous and fragmented. No identified instrument provides a comprehensive, multidisciplinary assessment of neurotrauma capacity across the full continuum of care. These findings support the development of an integrated framework combining indicators across the neurotrauma care pathway.

## Highlights

• Comprehensive mapping of 19 neurotrauma capacity instruments.
• No existing tool captures all neurotrauma care domains.
• Prehospital and workforce domains are consistently underrepresented.
• A dedicated neurotrauma framework is needed for health system strengthening.

## 1 Introduction

Neurotrauma, comprising traumatic brain injury (TBI) and traumatic spinal cord injury (tSCI), remains a major global health issue, with low- and middle-income countries (LMICs) bearing a disproportionately high burden of morbidity and mortality (Dewan et al., 2018; Roozenbeek et al., 2013; Lu et al., 2025). Outcomes are influenced not only by patient and injury characteristics, but also by the capacity of health systems and institutions to provide timely and appropriate care (Menon et al., 2026; Clark et al., 2022). Neurotrauma requires a coordinated pathway spanning prehospital care, emergency assessment, imaging, surgery, neurocritical care, and rehabilitation (Shammassian et al., 2025). This is particularly challenging in low-resource settings, where the absence of standardized protocols and coordinated systems may impede timely intervention and continuity of care (Roach et al., 2026). In such contexts, having the necessary equipment or personnel is not sufficient if these resources are not available when needed and appropriately integrated within the care pathway (Mock et al., 2026).

Despite this recognized need for integrated and multidisciplinary capacity, current assessment frameworks often fail to capture these dimensions. Indeed, most existing instruments are frequently limited by a lack of subspecialty specificity. In general, surgical or trauma indicators or checklists provide a broad overview of facility resources but mostly fail to account for the unique requirements of neurotrauma (World Health Organization (WHO), 2007; Groen et al., 2012; Kwon et al., 2012). Furthermore, such tools often prioritize the actual presence of resources over their accessibility.

This gap creates significant structural barriers in global health research and policy. Specifically, it limits meaningful benchmarking between institutions, affects the interpretation of clinical outcomes, and hinders the ability of policymakers to direct targeted investments toward identified systemic bottlenecks. Moreover, while the development of resource-stratified clinical protocols is an emerging approach for improving management in diverse settings, their successful implementation requires a rigorous, data-driven diagnosis of a facility’s actual capabilities.

This scoping review aims to map the instruments currently available to evaluate trauma and neurosurgical capacity across diverse global settings.

Unlike previous reviews of surgical and trauma capacity assessments, which have primarily focused on evaluating existing instruments and methodological approaches to conducting capacity assessments, the present review specifically examines the extent to which available frameworks capture the multidisciplinary and time-dependent requirements of neurotrauma across the continuum of care, including both capacity and quality-related domains (Blair et al., 2017).

This effort aims to identify gaps and guide the development of more precise institutional assessment frameworks for neurotrauma care across diverse settings.

## 2 Methods

### 2.1 Study design and reporting standards

This scoping review was conducted in accordance with the Joanna Briggs Institute (JBI) methodology for scoping reviews and is reported following the Preferred Reporting Items for Systematic Reviews and Meta-Analyses extension for Scoping Reviews (PRISMA-ScR) guidelines (Aromataris et al., 2019; Tricco et al., 2018). The review protocol was developed a priori by the two lead authors (NM and DB), who jointly defined the research question, eligibility criteria, search strategy, and outcomes of interest. The protocol was prospectively registered on the Open Science Framework (OSF) (OSF registration). The objective of this scoping review was to systematically map existing instruments designed to assess trauma and neurosurgical capacity, with specific attention to the extent to which these tools capture indicators essential for neurotrauma care at the local system and facility level.

### 2.2 Research question

The following research question guided the search strategy, study selection, data extraction, and synthesis:

*“What instruments are currently available to evaluate trauma and neurosurgical capacity across diverse global settings, and to what extent do these tools capture local system and facility-level indicators relevant to neurotrauma care?”*

For our purposes, capacity was defined following the framework of the Lancet Commission on Global Surgery as *the ability of a health system or healthcare facility to deliver safe, timely, and affordable care through adequate human resources, infrastructure, equipment, supplies, and governance* (Meara et al., 2015).

### 2.3 Eligibility criteria

The review followed the Population-Concept-Context (PCC) framework recommended for scoping reviews:

Population: We included assessment instruments, checklists, frameworks, or structured sets of indicators designed to evaluate system, institutional or facility-level capacity to deliver care. Eligible sources included peer-reviewed publications as well as grey literature. Studies describing or applying such tools in the evaluation of trauma, or neurosurgical care delivery were eligible for inclusion.

Concept: The concept of interest was the measurement of system or facility-level capability to manage neurotrauma patients. We included tools assessing any domain relevant to neurotrauma care, including but not limited to: prehospital and referral systems, emergency department readiness, imaging availability, surgical and operative capacity, neurocritical care and monitoring, nursing specialization, workforce training, referral pathways, and quality improvement or audit systems. Quality indicator sets were included because they frequently contain structure- or process-related indicators relevant to facility-level capacity evaluation. We excluded instruments focused exclusively on patient-level outcomes, clinical prognostic scores, disease severity classifications, or individual clinician performance, as these do not assess institutional capacity.

Context: We included tools designed for or applied in any healthcare facility setting globally, across all income levels and health system structures. Attention was given to instruments developed for, validated in, or applied within LMICs context. No restrictions were applied on the year of publication. Studies published in any language were considered for inclusion where translation in the English language was feasible by standard online tools (ie Google Translate®).

### 2.4 Search strategy

The following databases were searched from inception to March 06, 2026: PubMed/MEDLINE, Embase, Web of Science, and Global Health. Grey literature was searched through World Health Organization (WHO) IRIS, ReliefWeb, and Google Scholar. The search strategy combined controlled vocabulary terms (e.g., MeSH) and free-text keywords structured around three core conceptual blocks:

1. Assessment and measurement
2. Care setting or system
3. Clinical domain

The search strategy was developed along with a professional librarian and executed by one author (NM) and independently cross-checked by a second author (DB). Search strategies were adapted by the librarian to each database (Supplement 1). Pediatric exclusion filters were applied to the primary database search to streamline initial screening. Reference mining of included studies and grey literature were conducted without age-related restrictions to ensure comprehensive capture of all relevant facility-level assessment tools, regardless of target population. The systematic search was prespecified to end on March 6, 2026, and this date was strictly applied as the formal search cutoff. Therefore, the updated second edition of the WHO Guidelines for Essential Trauma Care, which became available after the prespecified search cutoff, was not included in the formal instrument selection or domain mapping. It was subsequently considered qualitatively to provide relevant contextual information and to assess whether the updated guidance addressed limitations identified in the included instruments (Mock et al., 2026).

### 2.5 Study selection

All search results were imported into the Rayyan® web-based platform for reference management and screening, and duplicate records were removed. Title and abstract screening was conducted independently by two reviewers (NM and DB). Full texts of potentially eligible studies were then independently reviewed by the same two reviewers. Disagreements at any stage were resolved through discussion and consensus. Reference lists of included studies and relevant review articles were manually screened to identify additional eligible sources.

### 2.6 Data extraction

Data were extracted using a standardized form, which was piloted on five included studies and refined iteratively.

The six domains used for the neurotrauma capacity mapping were predefined a priori based on the expected continuum of neurotrauma care and on the domains represented across the capacity-assessment literature: (1) prehospital and referral systems, (2) emergency and imaging readiness, (3) operative capacity, (4) neurocritical care and monitoring, (5) workforce and training, and (6) quality and audit systems. Each item or indicator within an included instrument was assigned to the domain that best represented its primary function. Items addressing more than one domain were assigned according to their principal purpose to avoid double-counting. Domain coverage was subsequently coded as present when at least one item within the instrument was mapped to that domain.

Extraction was performed independently by two reviewers (NM and DB), with cross-checking for accuracy and completeness.

### 2.7 Data synthesis and reporting

Extracted data were synthesized descriptively and presented in tabular and narrative formats. Instruments were categorized according to their primary scope. A thematic mapping approach was used to identify which domains of neurotrauma care were or not assessed. This mapping was used to highlight structural gaps in existing instruments and to inform the generation of candidate indicators for subsequent development of a neurotrauma capacity framework.

The study selection process was documented in sufficient detail to generate a PRISMA flow diagram, and a list of excluded full-text studies with reasons for exclusion is provided.

### 2.8 Bias assessment

Consistent with methodological guidance for scoping reviews, a formal risk of bias or quality assessment was not undertaken, as the objective of the review was to map the breadth, characteristics, and content of existing assessment instruments rather than to evaluate the quality of evidence or estimate effects.

### 2.9 Statistical analysis

Given the descriptive and exploratory nature of this scoping review, no quantitative synthesis or meta-analysis was performed.

### 2.10 Ethics and dissemination

No ethical approval was required, as this review is based on already published data and did not involve interaction with human subjects.

## 3 Results

### 3.1 Overview of the included studies

The initial literature search identified 12,424 records. After deduplication (n = 4008), 8416 unique records were screened. Full-text assessment was performed for 36 articles, of which 22 were excluded (Supplement 2), leaving 14 documents from the primary database search. Additional reference mining and grey literature searches identified a further 12 documents. Overall, a total of 26 documents were included in the final data extraction and synthesis (Fig. 1).

**Fig. 1:**
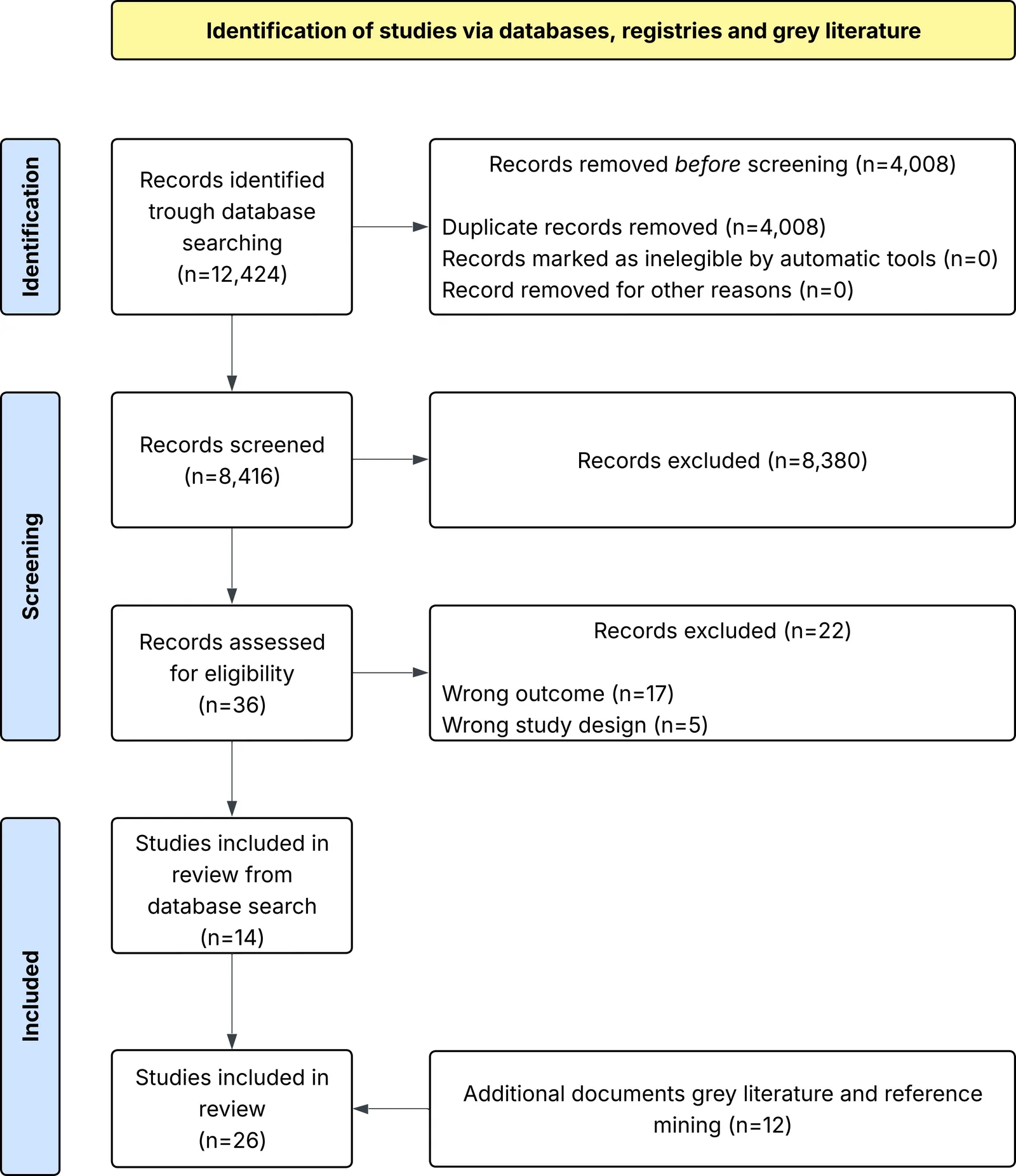
PRISMA flow diagram of the study selection process. After deduplication, 8416 records were screened. Thirty-six full-text articles were assessed; 22 were excluded (Supplement 2). A total of 26 documents were included in the final synthesis (14 from databases, 12 from supplementary sources).

Publication dates ranged from 2004 to 2026, with the majority (n = 19; 73%) published after 2015. Geographically, studies were conducted across a broad range of settings. Sixteen studies (62%) were conducted exclusively in LMICs while 7 studies (27%) were in high-income countries (HICs). Two studies (8%) had a global or multi-country scope encompassing both HIC and LMIC settings. One additional WHO technical document (4%) was developed specifically for LMIC contexts but without a single-country focus.

Regarding study design, cross-sectional surveys and facility assessments (n = 11; 42%) were the most common, followed by Delphi consensus studies (n = 7; 27%) and instrument development or adaptation studies (n = 5; 19%).

In terms of purpose, studies broadly fell into three categories: capacity assessment and tool development (n = 13; 50%), quality indicator development (n = 9; 35%), and instrument validation or adaptation (n = 4; 15%). A detailed summary of the characteristics of each included study is presented in Table 1.

**Table 1:** Characteristics of the included studies. The table summarizes key characteristics of all documents included in the scoping review, organized by first author and year. Studies are presented with their geographic setting, income classification, study design, target facility level, and primary study purpose. Income classification follows the World Bank definition at the time of each study. Abbreviations: LMIC, low- and middle-income country; HIC, high-income country; TBI, traumatic brain injury; QIs, quality indicators; msTBI, moderate-to-severe traumatic brain injury; NCC, neurocritical care; EESC, emergency and essential surgical care.

Author (year)	Country/Region	Income setting	Study design	Study purpose
Adusumilli et al. (2025)	India	LMIC	Cross-sectional	Assess TBI preparedness
Baker et al. (2013)	Tanzania	LMIC	Cross-sectional	Assess emergency/critical care resources
Barkley et al. (2019)	Cambodia	LMIC	Mixed-methods	Assess TBI care capacity
Bhatia et al. (2021)	India	LMIC	Cross-sectional	Assess surgical/trauma capacity
Bieler et al. (2020)	Germany	HIC	Expert consensus	Select trauma quality indicators
Blair et al. (2017)	Bolivia	LMIC	Cross-sectional	Assess surgical/trauma capacity
Coccolini et al. (2021)	International	Mixed	Systematic review + Consensus	Develop global trauma QIs
Groen et al. (2012)	Sierra Leone	LMIC	Cross-sectional/Tool development	Develop and validate PIPES
Huijben et al. (2019)	Europe (18 countries)	HIC	Delphi consensus	Develop TBI ICU QIs
Jeffcote et al. (2025)	Australia	HIC	Delphi consensus	Develop msTBI management QIs
Kingham et al. (2009)	Sierra Leone	LMIC	Cross-sectional	Assess surgical capacity
Kovoor et al. (2022)	Australia/New Zealand	HIC	Lit review + Expert working group	Propose novel trauma QIs
Kwon et al. (2012)	Sierra Leone, Liberia, Solomon Islands	LMIC	Cross-sectional/Index development	Develop surgical capacity index
Markin et al. (2014)	Bolivia	LMIC	Validation study	Validate PIPES reliability
Mock et al. (2004)	Global	Mixed	Expert consensus/Guidelines	Define essential trauma care standards
Moheet et al. (2018)	United States	HIC	Expert consensus/Standards	Establish NCC unit standards
Moore et al. (2025)	Canada, US, UK, Australia, Sweden	HIC	RAND/UCLA Delphi	Develop pediatric trauma QIs
Moore et al. (2026)	Canada	HIC	Retrospective cohort validation	Validate pediatric trauma QIs
Mousazadeh et al. (2022)	Iran	LMIC	Multi-method (Lit review + Delphi)	Develop trauma performance model
Notrica et al. (2011)	Rwanda	LMIC	Cross-sectional	Assess surgical/anesthesia infrastructure
Ploss et al. (2017)	Uganda	LMIC	Cross-sectional/Tool development	Develop Neuro-PIPES tool
Stewart et al. (2016) a	Ghana	LMIC	Cross-sectional (serial)	Assess trauma capacity over time
Stewart et al. (2016) b	Ghana	LMIC	Delphi consensus	Develop trauma audit filters
Ukachukwu et al. (2024)	Nigeria	LMIC	Cross-sectional	Assess neurosurgical capacity
Wong et al. (2014)	Sierra Leone	LMIC	Retrospective/Tool development	Develop INTACT index
WHO (2007)	Global	LMIC-focused	Instrument development	Develop EESC assessment tool

### 3.2 Instruments identified

A total of 19 distinct instruments were identified and categorized according to their primary focus. Four instruments were designed to assess general surgical capacity. The WHO Tool for Situational Analysis to Assess Emergency and Essential Surgical Care (TSAAEESC) is a comprehensive 256-item questionnaire covering infrastructure, human resources, interventions, and equipment, designed for first-referral level facilities (World Health Organization (WHO), 2007; Kingham et al., 2009). The personnel, infrastructure, procedures, equipment and supplies (PIPES) tool, adapted from the WHO TSAAEESC, comprises 105 items across five domains and generates a normalized composite index from 0 to 10. The Surgical Capacity Scoring Index applies weighted scoring to WHO TSAAEESC data to generate a 0–100 index with four capacity levels (Kwon et al., 2012). The Harward Humanitarian Initiative (HHI) tool assesses surgical and anesthesia infrastructure across eight domains but does not include a formal scoring system (Notrica et al., 2011).

Four instruments focused specifically on trauma care capacity. The WHO Guidelines for Essential Trauma Care provide 260 items across 14 categories with a 0–3 rating scale and four-level prioritization (Essential/Desirable/Possibly Required/Irrelevant), explicitly including sections on head and spinal injury (Mock et al., 2004). The International Assessment of Capacity for Trauma (INTACT) index is a trauma-specific adaptation of PIPES, comprising 40 binary items with a 0–10 index (Wong et al., 2014). The Emergency and Critical Care (EaCC) tool assesses emergency and critical care resources across 104 indicators using a percentage-based scoring system (Baker et al., 2013). The Performance Assessment Model provides a framework with 50 indicators across six components, though it does not specify a formal scoring system (Mousazadeh et al., 2022).

Four instruments focused specifically on neurosurgical or neurocritical care capacity. Neuro-PIPES, an adaptation of PIPES, adds 21 neurosurgery-specific items to generate a 0–10 index (Total/126 × 10) (Ploss et al., 2017). The Modified Neuro-PIPES expands this further to 180 items across five domains, maintaining the same binary scoring and 0–10 index (Ukachukwu et al., 2024). The Neurocritical Care (NCC) Checklist, developed by the Neurocritical Care Society, provides a 261-item standards document with a 3-tier classification (Levels I, II, and III) for neurocritical care units, covering organization, personnel, equipment, and training (Moheet et al., 2018). The Level-numeric TBI scoring system was developed specifically for the Indian context, generating a 4-level classification and numeric score (0–53) based on facilities, human resources, and intensive care unit (ICU) capabilities (Adusumilli et al., 2025).

Seven instruments were quality indicator sets for trauma or neurotrauma care (Bieler et al., 2020; Coccolini et al., 2021; Huijben et al., 2019; Jeffcote et al., 2025; Kovoor et al., 2022; Moore et al., 2025; Stewart et al., 2016). All indicator sets employ benchmarking approaches without generating composite scores, instead providing process and outcome measures for quality improvement and audit purposes.

A detailed summary of the characteristics of each instrument, organized by primary focus, is presented in Table 2.

**Table 2:** Characteristics of included instruments. Instruments are categorized by primary focus: surgical capacity, trauma capacity, neurosurgical and neurocritical care capacity, and quality indicator sets. The table summarizes each instrument’s type, specificity, target setting, and scoring system. For instruments using an index scoring system (PIPES, Neuro-PIPES, MN-PIPES, INTACT), the score represents the proportion of items present multiplied by 10, ranging from 0 to 10, with higher scores indicating greater capacity. E/D/PR/I, Essential/Desirable/Possibly Required/Irrelevant; LMIC, low- and middle-income country; TBI, traumatic brain injury; msTBI, moderate-to-severe traumatic brain injury; NCC, neurocritical care; QIs, quality indicators.

Instrument name	Reference	Type	Primary focus	Specifics	Target setting	Scoring system
**Surgical Capacity**						
WHO TSAAEESC	WHO (2007)	Checklist/Questionnaire	Surgical capacity	Emergency & essential surgical care	District/first-referral hospitals	Subjective rating (always/sometimes/never; provided/referred; absent/shortages/fully available)
PIPES	Groen et al. (2012)	Checklist/Questionnaire	Surgical capacity	General surgery	District to tertiary hospitals	Binary (1/0); Index (0-10)
Surgical Capacity Scoring Index	Kwon et al. (2012)	Index/Scoring system	Surgical capacity	Derived from WHO TSAAEESC	District to tertiary hospitals	Weighted scoring; Index (0-100); 4-level classification
HHI tool	Notrica et al. (2011)	Checklist/Questionnaire	Surgical/Anesthesia capacity	Infrastructure, personnel, equipment	District hospitals	None (survey format)
**Trauma Capacity**						
INTACT	Wong et al. (2014)	Checklist/Questionnaire	Trauma capacity	Trauma-specific; derived from PIPES	District to tertiary hospitals	Binary (1/0); Index (0-10)
WHO EsTC	Mock et al. (2004)	Checklist/Standards	Trauma capacity	Essential trauma care; includes neurotrauma	Basic to tertiary facilities	0-3 scale; 4-level prioritization (E/D/PR/I)
EaCC tool	Baker et al. (2013)	Checklist/Standards	Emergency/Critical Care	Emergency & critical care resources	District to referral hospitals	Percentage of items present
Performance Assessment Model	Mousazadeh et al. (2022)	Model/Framework + Indicators	Trauma care performance	Trauma care (traffic injuries)	Trauma centers	Not fully specified
**Neurosurgical & Neurocritical Care Capacity**						
Neuro-PIPES	Ploss et al. (2017)	Checklist/Questionnaire	Neurosurgical capacity	Neurosurgery-specific; retains PIPES comparability	Neurosurgical centers	Binary (1/0); Index (0-10)
MN-PIPES	Ukachukwu et al. (2024)	Checklist/Questionnaire	Neurosurgical capacity	Modified Neuro-PIPES (expanded)	Neurosurgical centers	Binary (1/0); Index (0-10)
NCC Checklist	Moheet et al. (2018)	Checklist/Standards	Neurocritical care	NCC unit standards (Levels I, II, III)	Neurocritical Care Units	3-tier classification (Level I, II, III)
Level-numeric TBI scoring	Adusumilli et al. (2025)	Criteria/Scoring system	TBI capacity	TBI preparedness	Secondary to tertiary hospitals	4-level classification + numeric score (0-53)
**Quality Indicator Sets**						
43 TR-DGU QIs	Bieler et al. (2020)	Quality Indicator Set	Trauma quality	TraumaRegister DGU; 13 selected	Trauma centers	Not applicable (benchmarking)
82 Trauma QIs	Coccolini et al. (2021)	Quality Indicator Set	Trauma quality	International consensus; 6 categories	Trauma centers globally	Not applicable (benchmarking)
42 TBI ICU QIs	Huijben et al. (2019)	Quality Indicator Set	TBI quality	TBI in ICU; 13 clinical domains	ICUs (European)	Not applicable (benchmarking)
32 msTBI QIs	Jeffcote et al. (2025)	Quality Indicator Set	TBI management quality	Acute msTBI management	Australian neurotrauma centers	Not applicable (benchmarking)
31 Novel Trauma QIs	Kovoor et al. (2022)	Quality Indicator Set	Trauma quality	Proposed novel indicators	Trauma centers	Not applicable (benchmarking)
23 Pediatric Trauma QIs	Moore et al. (2025)	Quality Indicator Set	Pediatric trauma quality	Pediatric-specific evidence	Pediatric trauma centers	Not applicable (benchmarking)
22 Audit Filters	Stewart et al. (2016)	Audit Filters	Trauma quality/audit	LMIC district hospitals; context-appropriate	District hospitals (LMIC)	Not applicable (process measures)

### 3.3 Domain coverage and gap analysis

The 19 instruments were mapped across six predefined domains reflecting the main components of neurotrauma care: prehospital and referral systems, emergency and imaging readiness, operative capacity, neurocritical care and monitoring, workforce and training, and quality and audit systems (Table 3). This mapping reflects a broad-level assessment of domain presence: a “Yes” indicates the presence of at least one item addressing that domain, but does not imply comprehensive coverage. A detailed item-level content analysis was beyond the scope of this review, and readers are referred to the original instruments for comprehensive item lists. Items were assigned to a single domain according to their primary function, with domain presence defined by the inclusion of at least one mapped item.

**Table 3:** Domain coverage of neurotrauma-relevant capacity across included instruments. The table maps coverage of six neurotrauma-relevant domains across instruments, grouped by primary focus. “Yes” indicates the presence of at least one item relevant to that domain; “No” indicates that no items addressing that domain were identified. This mapping is intended to identify broad patterns of coverage and gaps across existing instruments. A “Yes” indicates presence of at least one item in this domain but does not imply comprehensive coverage. Readers should consult original instruments for detailed item-level content.

Instrument name	Pre-hospital & referral	ED & Imaging	Operative Capacity	Neurocritical Care	Workforce & Training	Quality & Audit
**Surgical Capacity**						
WHO TSAAEESC	no	yes	no	no	no	yes
PIPES	no	yes	no	no	no	no
Surgical Capacity Scoring Index	no	no	no	no	no	yes
HHI tool	no	no	no	no	no	yes
**Trauma Capacity**						
INTACT	no	yes	no	no	no	no
WHO EsTC	yes	yes	yes	yes	yes	yes
EaCC tool	no	yes	no	no	no	no
Performance Assessment Model	no	yes	no	no	no	yes
**Neurosurgical & Neurocritical Care Capacity**						
Neuro-PIPES	no	yes	yes	no	yes	no
MN-PIPES	no	yes	yes	yes	yes	no
NCC Checklist	no	yes	no	yes	yes	yes
Level-numeric TBI scoring	no	yes	yes	yes	yes	no
**Quality Indicator Sets**						
43 TR-DGU QIs	yes	yes	yes	yes	no	yes
82 Trauma QIs	yes	yes	yes	yes	no	yes
42 TBI ICU QIs	no	yes	yes	yes	yes	yes
32 msTBI QIs	yes	yes	yes	yes	yes	yes
31 Novel Trauma QIs	yes	yes	yes	no	no	yes
23 Pediatric Trauma QIs	yes	yes	yes	yes	no	yes
22 Audit Filters	yes	yes	no	no	no	yes

Emergency and imaging readiness was the most frequently covered domain, with 17 instruments (89%) including at least one item. This was followed by quality and audit systems (n = 13; 68%), operative capacity (n = 10; 53%), and neurocritical care and monitoring (n = 9; 47%). The least covered domains were prehospital and referral systems and workforce and training, each addressed by only seven instruments (37%).

Coverage patterns differed across instrument types. Among surgical and trauma capacity tools (n = 8), coverage was primarily concentrated in emergency and imaging readiness and quality and audit systems, while operative capacity, neurocritical care, workforce, and prehospital domains were addressed only exceptionally (largely through the WHO Essential Trauma Care Guidelines). In contrast, neurosurgical and neurocritical care tools (n = 4) consistently covered emergency and imaging readiness, workforce, and - to a lesser extent-operative and neurocritical care domains, without addressing prehospital and referral systems. Quality indicator sets (n = 7) demonstrated the most balanced coverage across domains, although workforce and training remained underrepresented even in this group.

### 3.4 Methodological characteristics

The development methods of the 19 included instruments varied considerably (Table 4). Instruments could be broadly categorized into two groups: those developed through formal consensus methodologies (n = 10, 53%) and those developed through informal expert consultation or author consensus (n = 9; 47%).

**Table 4:** Methodological features of the included instruments. The table summarizes the development methods (including expert panel composition where reported) and key strengths and limitations for each included instrument. Instruments are grouped by primary focus. LMIC, low- and middle-income country; HIC, high-income country; QIs, quality indicators.

Instrument name	Development Method	Key strengths	Key limitations
**Surgical Capacity**			
WHO TSAAEESC	WHO expert consensus (multiple experts)	Comprehensive; widely used (≥35 countries)	Subjective; 256 items; no formal index
PIPES	Adaptation of WHO TSAAEESC	Simple; binary scoring; repeatable	Not neuro-specific; no operational readiness
Surgical Capacity Scoring Index	Quantitative scoring allocated to WHO TSAAEESC items	Quantitative index; 4-level classification; cross-country application	Weighted scoring arbitrary; no validity testing
HHI tool	Adaptation of existing tools	Covers 8 domains; district-level focus	No formal scoring system; not neuro-specific
**Trauma Capacity**			
INTACT	selection of items fromWHO TSAAEESC and PIPES; Group consensus (3 authors)	Trauma-specific; correlates with PIPES	Limited consensus; no Delphi; small sample
WHO EsTC	WHO/IATSIC expert consensus (multiple experts + 14 consultation)	Global applicability; includes neurotrauma sections	Subjective; 260 items; no formal scoring
EaCC tool	Adaptation of WHO TSAAEESC (∼16 experts)	Developed for LMIC; comprehensive	No formal Delphi; limited validation
Performance Assessment Model	Lit review + 2-round Delphi + focus groups + interviews (30 experts)	Multi-method; LMIC-specific; includes patient satisfaction	Single-center pilot; no reliability testing
**Neurosurgical & Neurocritical Care Capacity**			
Neuro-PIPES	Adaptation of PIPES + expert panel (5 experts)	Neuro-specific; retains PIPES comparability; reliability assessed	Small panel; limited reliability for equipment/supplies
MN-PIPES	Adaptation of Neuro-PIPES	Large national sample (50 facilities)	No reliability/validity testing
NCC Checklist	NCS expert consensus; multidisciplinary writing group	Comprehensive NCC standards; 3-tier levels; multidisciplinary	HIC-based; 261 items; no formal validation
Level-numeric TBI scoring	Lit review + expert panel content validation (5 experts; single round)	Simple; context-specific; feasible; content validity assessed	No reliability testing; small sample
**Quality Indicator Sets**			
43 TR-DGU QIs	QUALIFY methodology; expert meeting (12 experts)	Systematic QUALIFY methodology; 13 quality criteria	HIC-only; 0/43 structure indicators
82 Trauma QIs	Lit review + 2-round Delphi + international meeting (200 experts)	Largest global QI study; 5 continents; neuro-specific indicators	Only 2/82 structure indicators; no formal validation
42 TBI ICU QIs	3-round Delphi (50 experts)	Rigorous 3-round Delphi; large European panel; TBI-specific	No LMIC; no nurses; no patients
32 msTBI QIs	Advisory Committee + modified Delphi (43 experts; single round)	Nationally representative; structured by care pathway	No caregivers; no rehabilitation clinicians; HIC-specific
31 Novel Trauma QIs	Lit review + expert working group (5 experts)	Based on systematic lit review; Donabedian framework	Small group; no formal consensus; not validated
23 Pediatric Trauma QIs	Modified RAND/UCLA Delphi (56-59 experts; 2 rounds)	Rigorous RAND/UCLA; caregivers included; pediatric-specific	HIC-only; limited geographic diversity
22 Audit Filters	4-round Delphi (20 experts)	Rigorous 4-round Delphi; 100% participation; LMIC-context	Developed primarily for Ghana; acts as process proxy rather than outcome tracker

Among the 10 instruments developed using formal consensus methodologies, methods included Delphi (ranging from one to four rounds; n = 6), RAND/UCLA methodology (n = 1), QUALIFY methodology (n = 1), and WHO-organized expert consensus (n = 2). Panel sizes ranged from 5 to 200 participants. Panel composition varied across instruments. Most panels consisted predominantly of physicians (neurosurgeons, intensivists, trauma surgeons, anesthesiologists and emergency physicians). Nurses were included in the development of the NCC Checklist and the 82 Trauma Quality Indicators but were absent from most other panels. Patient or caregiver involvement was reported only in the development of the 23 Pediatric Trauma Quality Indicators. Multidisciplinary panels - including professionals from multiple specialties - were more common among quality indicator sets than among capacity assessment tools.

## 4 Discussion

This scoping review identified 19 frameworks for assessing surgical, trauma, neurosurgical, or neurocritical care capacity. The instruments were heterogeneous in scope, methodology, and content. Most focused on general surgical or trauma, with only four specifically addressing neurosurgical or neurocritical care domains. No instrument covered all domains of neurotrauma capacity. Crucially, no instrument identified was designed specifically to assess neurotrauma capacity as a standalone construct (Fig. 2).

**Fig. 2:**
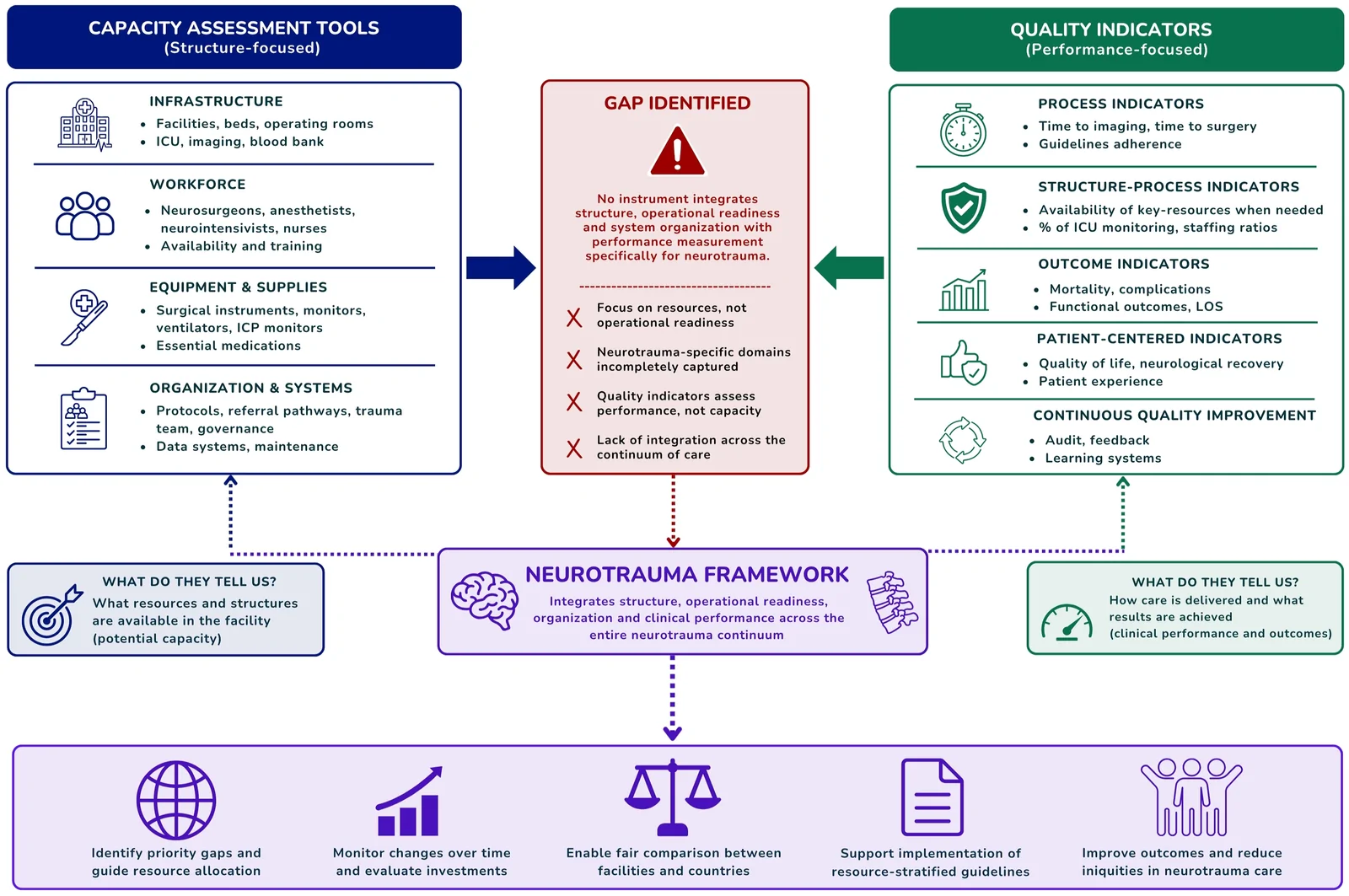
Conceptual framework of neurotrauma capacity assessment and quality improvement. Left: capacity assessment tools evaluate structural resources (infrastructure, workforce, equipment, organization). Right: quality indicators evaluate clinical performance (process, outcomes, patient-centered measures). Center: the proposed integrated framework combines both dimensions across the neurotrauma continuum to guide resource allocation, benchmarking, and guideline implementation. The central “GAP IDENTIFIED” box explicitly highlights the current absence of a unified instrument that integrates structural capacity assessment with quality indicators specifically for neurotrauma care, thereby underscoring the need for the proposed framework.

The lack of a specific tool for measuring neurotrauma is noteworthy, considering the global impact of these conditions. Indeed, TBI affects an estimated 27-69 million people annually, with the majority of cases in LMICs, where mortality is three-to four-fold higher than in HICs1,2. A similar disparity exists for tSCI, which affects 45-80 persons per million annually and is disproportionately concentrated in LMICs3. A recent multinational review of LMIC institutions found that only 41% reported the presence of neurosurgical capacity, and that implementation of standardized clinical protocols was significantly associated with increased neurosurgical capacity and reduced post-TBI mortality7. A global survey of 159 centers across 57 countries further documented profound disparities in resources across the TBI care pathway, with the largest gaps observed in prehospital and rehabilitation services4.

Without a standardized instrument to assess institutional capacity, it remains difficult to identify where limitations in neurotrauma care occur. Neurotrauma is a time-sensitive continuum: patients may require prehospital recognition and transport, emergency assessment and imaging, timely neurosurgical intervention, neurocritical care, and coordinated rehabilitation. A limitation at any of these stages may affect the effectiveness of subsequent care. General surgical or trauma capacity tools do not fully capture these neurotrauma-specific requirements.

Among the instruments identified, several demonstrated notable strengths. Tools such as PIPES and INTACT offer simple binary scoring and repeatability, enabling comparisons over time and across facilities10,20. The WHO Guidelines for Essential Trauma Care provide comprehensive coverage across facility levels and include dedicated sections on head and spinal injury19. It must be mentioned that since the completion of the literature search for this review, the second edition of the WHO Guidelines for Essential Trauma Care has been published8. Although this updated document was not eligible for inclusion in the present review, it reinforces several observations arising from our findings. The revised guidelines place greater emphasis on operational readiness, continuous availability of resources, equipment maintenance, continuing professional development, and quality improvement systems, confirming that effective trauma care depends not only on the presence of infrastructure but also on its functionality and integration within the health system (Mock et al., 2026; Corley et al., 2019). Furthermore, the updated resource matrix introduces a distinction between core and extended resources according to facility level, providing a more pragmatic framework for resource prioritization across diverse settings. Nevertheless, despite these important advances, the updated WHO Guidelines remain focused on essential trauma care rather than neurotrauma specifically. Quality indicator sets offer rigorous, consensus-based process and outcome measures for trauma care, with large, multidisciplinary expert panels (Coccolini et al., 2021; Huijben et al., 2019; Jeffcote et al., 2025). However, several shortcomings limit their utility for neurotrauma capacity assessment. First, these instruments focus on clinical performance rather than structural capability; they measure what is done (e.g., time to imaging, guideline adherence) rather than what resources are available to enable that care. While some sets include structure-related items (e.g., availability of neurocritical care beds, staffing ratios), these remain limited and rarely address operational readiness or functional accessibility. Second, neurotrauma-specific items are largely absent from most trauma quality indicator sets. Third, quality indicator sets, while methodologically robust, are designed primarily for benchmarking and quality improvement rather than capacity assessment, and have been developed predominantly in HIC settings, limiting their applicability to LMICs. Indeed, a fundamental distinction must be highlighted between capacity assessment tools and quality indicator sets (Fig. 2). The first (e.g., PIPES, WHO TSAAEESC, and Neuro-PIPES) are designed to evaluate what resources and structures exist within a facility. The latter assess processes and outcomes. Both are necessary, but they serve different purposes.

A hospital cannot be expected to perform well on quality indicators if it lacks the fundamental capacity to deliver care. Yet, as summarized in Fig. 2, the results of this review highlight that no instrument currently integrates both dimensions for neurotrauma - capacity and quality-into a unified specific framework. This limits the ability to distinguish between gaps in structural capability and gaps in clinical performance.

Existing instruments also suffer from a fundamental limitation: most are resource inventories rather than measures of accessibility. They capture what is nominally available, not whether it is consistently accessible when needed. Neurotrauma care, however, depends on the interaction between multiple time-critical processes. For example, the availability of a CT scanner has little clinical value if imaging cannot be performed around the clock, if radiological interpretation is delayed, or if neurosurgical expertise is unavailable to act upon the findings. Likewise, operating theatre availability alone does not ensure timely intervention without dedicated anesthesia support, ICU capacity, and structured referral pathways. These interdependencies have been increasingly recognized within the broader concept of health system strengthening, which emphasizes that healthcare performance emerges from coordinated functioning across system “building blocks” rather than isolated structural elements (Everybody’s business; Kruk et al., 2018; Donabedian, 1988). Future neurotrauma capacity assessment frameworks should therefore move beyond simple resource inventories toward evaluating accessibility, integration of services, and the reliability of care pathways12.

The gaps identified in this review have particular significance for lower-resource settings. First, health systems strengthening efforts may not be systematically targeted; investments may be directed toward areas already adequately resourced while critical gaps remain unaddressed (Namakula et al., 2025). Second, the effectiveness of investments cannot be evaluated without objective baseline and follow-up measurements (Gupta et al., 2025). Third, hospital teams in LMICs may lack the data needed to advocate effectively for resources (Kluyts et al., 2026). Fourth, without capacity adjustment, clinical outcomes from LMICs centers might be compared unfavorably to HICs centers, perpetuating a narrative of “poor performance” rather than “structural constraint.” Lastly, the development of resource-stratified clinical protocols, an emerging approach for improving neurotrauma management in diverse settings, requires precise understanding of facility-level capabilities (Rubiano et al., 2020; Marchesini et al., 2025a, 2025b).

Other surgical subspecialties have addressed this issue through systematic phased approaches. For example, the Global Assessment in Pediatric Surgery (GAPS) tool was developed through a systematic three-phase methodology, specifically to assess pediatric surgical capacity in low-resource settings (Yousef et al., 2019, 2024a, 2024b). The authors found a critical gap in the possibility to measure pediatric surgery capacity and developed a standardized tool assessing resources, accessibility, impact and training. Additionally, the application of this tool has been proven to be effective in highlighting critical gaps and offering evidence to support national development strategies in specific settings (Inglin et al., 2025). These experiences demonstrate that subspecialty-specific capacity tools are not only feasible but can provide actionable data for policymakers supporting the development of national surgical strategies.

The findings of this review provide a strong rationale for the development of a dedicated neurotrauma capacity assessment framework (Fig. 2). This instrument, rather than replacing existing surgical or trauma assessment tools, should integrate their strengths while addressing the gaps identified in the present review. Specifically, it should encompass the entire continuum of care (both for TBI and tSCI), including prehospital systems, emergency and imaging readiness, operative capability, neurocritical care, rehabilitation pathways, workforce competencies, governance, and quality improvement mechanisms. Importantly, indicators should extend beyond the simple presence of resources to evaluate their accessibility, functionality, and integration into clinical pathways. The resulting instrument should remain sufficiently pragmatic to permit routine implementation across diverse health systems while allowing benchmarking between institutions and monitoring changes over time. Ultimately, a standardized tool could facilitate international comparisons, support resource allocation, inform implementation of resource-stratified clinical guidelines, and provide an essential adjustment variable for multicenter neurotrauma research.

Future work should include formal validation and feasibility testing of the proposed framework across diverse settings, including LMICs, to ensure its applicability and utility in routine practice. Potential barriers to implementation in LMIC settings include limited financial and human resources, competing health system priorities, insufficient training for data collection and use, and the need for contextual adaptation of indicators to local realities. These factors should be anticipated and addressed during the development and piloting phases.

## 5 Strengths and limitations

This review has several strengths. To our knowledge, it represents the first comprehensive synthesis of instruments designed to assess facility-level capacity relevant to neurotrauma care. The review followed established JBI methodology, was prospectively registered, included both peer-reviewed and grey literature, and systematically mapped instrument content across predefined neurotrauma domains. By examining not only the characteristics of each instrument but also their methodological development and domain coverage, this review provides a comprehensive overview of the current landscape of capacity assessment.

Some limitations should also be acknowledged. First, despite a comprehensive search strategy, some locally developed assessment tools or unpublished institutional instruments may not have been identified. Second, the domain mapping relied on qualitative interpretation of instrument content and therefore inevitably involved some degree of subjective judgment, despite the data extraction performed independently by two reviewers. Third, although our primary database search excluded pediatric-focused studies, we included one pediatric-specific quality indicator set identified through supplementary searches. This asymmetry represents a pragmatic trade-off between screening efficiency and comprehensive mapping. Fourth, this review did not formally assess measurement properties such as validity, reliability, or responsiveness because its objective was to map existing instruments rather than critically appraise their psychometric performance. Finally, because most identified instruments have been developed in specific geographical or healthcare contexts, caution is warranted when generalizing their applicability across different health systems.

## 6 Conclusions

Current instruments provide valuable approaches for assessing surgical, trauma, neurosurgical, and neurocritical care capacity, however none was specifically developed to measure neurotrauma capacity as a distinct construct. Existing tools incompletely capture the multidisciplinary, time-dependent, and systems-based nature of neurotrauma care, while quality indicator frameworks address clinical performance rather than structural capability. This review identifies important conceptual and methodological gaps that should inform the development of future assessment frameworks. A standardized neurotrauma capacity framework may have the potential to improve benchmarking, guide health system strengthening, facilitate implementation of resource-stratified protocols, and enhance interpretation of neurotrauma outcomes across diverse healthcare settings. Next steps should include a formal consensus process with multidisciplinary stakeholders and pilot testing in diverse settings, including LMICs, to refine and validate the framework before broader implementation.

## CRediT authorship contribution statement

Nicolò Marchesini: Conceptualization, Methodology, Investigation, Writing - original draft, Visualization. Dina Belhaj Youssef: Conceptualization, Methodology, Investigation, Writing - review & editing. Ellianne Dos Santos Rubio: Writing - review & editing. Edoardo Picetti: Writing - review & editing. Tariq Khan: Writing - review & editing. Corrado Iaccarino: Writing - review & editing. Andrès M. Rubiano: Supervision, Writing – review & editing.

All authors have approved the final version of the manuscript and agree to be accountable for all aspects of the work. All authors meet the ICMJE criteria for authorship.

## Declaration of generative AI and AI-assisted technologies in the manuscript preparation process

During the preparation of this work, the authors used ChatGPT for grammar checking/language refinement. After using this tool, the authors reviewed and edited the content as needed and take full responsibility for the content of the published article.

## Funding

This research did not receive any specific grant from funding agencies in the public, commercial, or not-for-profit sectors.

## Declaration of competing interest

The authors declare that they have no known competing financial interests or personal relationships that could have appeared to influence the work reported in this paper.
