# Supplementary material for: From trauma capacity to neurotrauma capacity: A scoping review of existing assessment frameworks
Source: Brain Spine. 2026 Oct 12;6:107474. doi: 10.1016/j.bas.2026.107474 (PMC13683447; doi:10.1016/j.bas.2026.107474)
Supplement: Supplementary file 1 [file mmc2.pdf]

articles

title	year	journal	authors	notes
Quality measures in spine surgery	2017	Spine	Bennett C. et al.	wrong study design
Nursing-sensitive Indicators for Quality Improvement for Patients with Traumatic Brain Injury	2024	Alternative therapies in health and medicine	Xu X. et al.	wrong outcome
The method of integral evaluation of the efficiency of the neurosurgical clinic based on the assessment of the degree of achievement of key performance indicators	2023	N.N. Priorov Journal of Traumatology and Orthopedics	Usachev D.Yu. et al.	wrong outcome
Quality Indicators for Children with Traumatic Brain Injury after Transition to an American College of Surgeons Level i Pediatric Trauma Center	2022	Pediatric Emergency Care	Campbell M. et al.	wrong outcome
What is the state of quality measurement in spine surgery?	2018	Clinical Orthopaedics and Related Research	Bennett C. et al.	wrong study design
Methodological comparisons in assessing neurosurgical capacity in uganda	2017	Annals of Global Health	Zick B. et al.	wrong outcome
Can quality be measured in neuroanaesthesia?	2012	European Journal of Anaesthesiology	Cowman S. et al.	wrong outcome
Assessment of quality of care using quality indicators: Study of a trauma centre registry	2010	Resuscitation	Macarenhas C. et al.	wrong outcome
Development of an instrument for measuring the quality of nursing care	1974	Nursing Research	Cornell S.A.	wrong outcome
Identifying Quality Indicators for the Care of Hospitalized Injured Older Adults: A Scoping Review of the Literature	2023	Journal of the American Medical Directors Association	Giroux, M et al.	wrong study design
Poverty of resource, abundance of cultural knowledge: Mokopuna Maori Traumatic Brain Injury and measurement tool development	2016	Journal of the American Academy of Child and Adolescent Psychiatry	Elder, H	wrong outcome
Evaluating Trauma Care Capacity and Capability in Low-Resource Conflict Settings: Pilot Implementation of the International Assessment of Capacity for Trauma-Blast Tool at Regional Health Facilities in Burkina Faso.	2026	World journal of surgery	Nacanabo YAR et al.	wrong outcome
Quantitative Assessment of Surge Capacity in Rwandan Trauma Hospitals: A Survey Using the 4S Framework.	2025	International journal of environmental research and public health	Velin L et al.	wrong outcome
Quality Indicators Targeting Low-Value Clinical Practices in Trauma Care.	2022	JAMA surgery	Moore L et al.	wrong outcome
A National Assessment of Trauma Systems Using the American College of Surgeons NBATS Tool: Geographic Distribution of Trauma Center Need.	2022	Annals of surgery	Dalton MK et al.	wrong outcome
Development of a novel Global Trauma System Evaluation Tool and initial results of implementation in the Republic of South Sudan.	2014	Injury	Remick KN et al.	wrong outcome
A comparison of methods to obtain a composite performance indicator for evaluating clinical processes in trauma care.	2013	The journal of trauma and acute care surgery	Moore L et al.	wrong outcome
Quality indicators used by trauma centers for performance measurement.	2012	The journal of trauma and acute care surgery	Santana MJ et al.	wrong outcome
Indicators of the quality of trauma care and the performance of trauma systems.	2012	The British journal of surgery	Gruen RL et al.	wrong outcome
Evidence for quality indicators to evaluate adult trauma care: a systematic review.	2011	Critical care medicine	Stelfox HT et al.	wrong study design
Quality indicators for evaluating trauma care: a scoping review.	2010	Archives of surgery (Chicago, Ill. : 1960)	Stelfox HT et al.	wrong study design
