# Supplementary material for: From trauma capacity to neurotrauma capacity: A scoping review of existing assessment frameworks
Source: Brain Spine. 2026 Oct 12;6:107474. doi: 10.1016/j.bas.2026.107474 (PMC13683447; doi:10.1016/j.bas.2026.107474)
Supplement: Supplementary file 2 [file mmc1.docx]

**PUBMED Search Strategy**

| Search | Query | Results |
| --- | --- | --- |
| #21 | Search: #20 NOT ("Animals"[Mesh] NOT "Humans"[Mesh]) | [3,](https://pubmed.ncbi.nlm.nih.gov/?term=%2320+NOT+%28%22Animals%22%5BMesh%5D+NOT+%22Humans%22%5BMesh%5D%29&sort=relevance&ac=no)805 |
| #20 | Search: #19 NOT ("Adolescent"[Mesh] OR "Child"[Mesh] OR "Infant"[Mesh] OR adolescen*[tiab] OR child*[tiab] OR schoolchild*[tiab] OR infant*[tiab] OR girl*[tiab] OR boy[tiab] OR boys[tiab] OR boyhood[tiab] OR teen[tiab] OR teens[tiab] OR teenager*[tiab] OR youth*[tiab] OR pediatr*[tiab] OR paediatr*[tiab] OR puber*[tiab]) | [3,854](https://pubmed.ncbi.nlm.nih.gov/?term=%2319+NOT+%28%22Adolescent%22%5BMesh%5D+OR+%22Child%22%5BMesh%5D+OR+%22Infant%22%5BMesh%5D+OR+adolescen%2A%5Btiab%5D+OR+child%2A%5Btiab%5D+OR+schoolchild%2A%5Btiab%5D+OR+infant%2A%5Btiab%5D+OR+girl%2A%5Btiab%5D+OR+boy%5Btiab%5D+OR+boys%5Btiab%5D+OR+boyhood%5Btiab%5D+OR+teen%5Btiab%5D+OR+teens%5Btiab%5D+OR+teenager%2A%5Btiab%5D+OR+youth%2A%5Btiab%5D+OR+pediatr%2A%5Btiab%5D+OR+paediatr%2A%5Btiab%5D+OR+puber%2A%5Btiab%5D%29&sort=relevance) |
| #19 | Search: #18 AND #3 AND #1 | [5,188](https://pubmed.ncbi.nlm.nih.gov/?term=%2318+AND+%233+AND+%231&sort=relevance&ac=no) |
| #18 | Search: ("Neurosurgery"[Mesh] OR "Craniocerebral Trauma"[Mesh] OR "Spinal Cord Injuries"[Mesh] OR "Brain Injuries, Traumatic"[Mesh] OR "trauma"[ti] OR "neuro-surg*"[ti] OR "neurosurg*"[ti] OR " neurotrauma*"[ti] OR "brain trauma*"[ti] OR "Neuro surg*"[ti] OR "neuro-trauma*"[ti] OR "traumatic brain injur*"[ti] OR TBI[ti] OR "spinal cord injur*"[ti] OR SCI[ti] OR "head injur*"[ti]) | [381,897](https://pubmed.ncbi.nlm.nih.gov/?term=%28%22Neurosurgery%22%5BMesh%5D+OR+%22Craniocerebral+Trauma%22%5BMesh%5D+OR+%22Spinal+Cord+Injuries%22%5BMesh%5D+OR+%22Brain+Injuries%2C+Traumatic%22%5BMesh%5D+OR+%22trauma%22%5Bti%5D+OR+%22neuro-surg%2A%22%5Bti%5D+OR+%22neurosurg%2A%22%5Bti%5D+OR+%22+neurotrauma%2A%22%5Bti%5D+OR+%22brain+trauma%2A%22%5Bti%5D+OR+%22Neuro+surg%2A%22%5Bti%5D+OR+%22neuro-trauma%2A%22%5Bti%5D+OR+%22traumatic+brain+injur%2A%22%5Bti%5D+OR+TBI%5Bti%5D+OR+%22spinal+cord+injur%2A%22%5Bti%5D+OR+SCI%5Bti%5D+OR+%22head+injur%2A%22%5Bti%5D%29&sort=relevance&ac=no) |
| #3 | Search: "Health Resources"[Mesh] OR "Staff Development"[Mesh] OR "Resource Allocation"[Mesh] OR "Workforce"[Mesh] OR "Personnel Management"[Mesh] OR "Quality Improvement"[Mesh] OR "Capacit*"[tiab] OR "Facilit*"[tiab] OR "Resource*"[tiab] OR "Workforce*"[tiab] OR "Personal manag*"[tiab] OR "Staff"[tiab] OR "Trauma center*"[tiab] OR "Treatment approach*"[tiab] OR "Quality Assurance*"[tiab] OR "quality improv*"[tiab] OR "Outcome and Process Assess*"[tiab] OR "quality improv*"[tiab] OR "Quality indicator*"[tiab] OR "quality assess* "[tiab] OR "quality measure* "[tiab] OR "quality control*"[tiab] | [3,176,519](https://pubmed.ncbi.nlm.nih.gov/?term=%22Health+Resources%22%5BMesh%5D+OR+%22Staff+Development%22%5BMesh%5D+OR+%22Resource+Allocation%22%5BMesh%5D+OR+%22Workforce%22%5BMesh%5D+OR+%22Personnel+Management%22%5BMesh%5D+OR+%22Quality+Improvement%22%5BMesh%5D+OR+%22Capacit%2A%22%5Btiab%5D+OR+%22Facilit%2A%22%5Btiab%5D+OR+%22Resource%2A%22%5Btiab%5D+OR+%22Workforce%2A%22%5Btiab%5D+OR+%22Personal+manag%2A%22%5Btiab%5D+OR+%22Staff%22%5Btiab%5D+OR+%22Trauma+center%2A%22%5Btiab%5D+OR+%22Treatment+approach%2A%22%5Btiab%5D+OR+%22Quality+Assurance%2A%22%5Btiab%5D+OR+%22quality+improv%2A%22%5Btiab%5D+OR+%22Outcome+and+Process+Assess%2A%22%5Btiab%5D+OR+%22quality+improv%2A%22%5Btiab%5D+OR+%22Quality+indicator%2A%22%5Btiab%5D+OR+%22quality+assess%2A+%22%5Btiab%5D+OR+%22quality+measure%2A+%22%5Btiab%5D+OR+%22quality+control%2A%22%5Btiab%5D&sort=relevance&ac=no) |
| #1 | Search: ("checklist*"[ti] OR "check-list*"[ti] OR "indicator*"[ti] OR "framework*"[ti] OR "tool*"[ti] OR "score*"[ti] OR " Evaluate*"[ti] OR "Screen*"[ti] OR "Instrument*"[ti] OR "scale"[ti] OR "Measure*"[ti] OR "Protocol"[ti] OR "survey*"[ti] OR "questionnair*"[ti] OR "interview*"[ti] OR "assess*"[ti] OR "Guideline*"[ti] OR "Index"[ti] OR "safet*"[ti]) | [2,432,252](https://pubmed.ncbi.nlm.nih.gov/?term=%28%22checklist%2A%22%5Bti%5D+OR+%22check-list%2A%22%5Bti%5D+OR+%22indicator%2A%22%5Bti%5D+OR+%22framework%2A%22%5Bti%5D+OR+%22tool%2A%22%5Bti%5D+OR+%22score%2A%22%5Bti%5D+OR+%22+Evaluate%2A%22%5Bti%5D+OR+%22Screen%2A%22%5Bti%5D+OR+%22Instrument%2A%22%5Bti%5D+OR+%22scale%22%5Bti%5D+OR+%22Measure%2A%22%5Bti%5D+OR+%22Protocol%22%5Bti%5D+OR+%22survey%2A%22%5Bti%5D+OR+%22questionnair%2A%22%5Bti%5D+OR+%22interview%2A%22%5Bti%5D+OR+%22assess%2A%22%5Bti%5D+OR+%22Guideline%2A%22%5Bti%5D+OR+%22Index%22%5Bti%5D+OR+%22safet%2A%22%5Bti%5D%29&sort=relevance&ac=no) |

**Embase Search Strategy**

1	("checklist*" or "check list*" or "indicator*" or "framework*" or "tool*" or "score*" or " Evaluate*" or "Screen*" or "Instrument*" or "scale" or "Measure*" or "Protocol" or "survey*" or "questionnair*" or "interview*" or "asses*" or "Guideline*" or "Index" or "safet*").ti.	3216362

2	*health care planning/ or *personnel management/ or *resource allocation/ or *resource management/ or *health workforce/ or *total quality management/ or *quality control/ or *health care quality/	245348

3	("Capacit*" or "Facilit*" or "Resource*" or "Workforce*" or "Personal manag*" or "Staff" or "Trauma center*" or "Treatment approach*" or "Quality Assurance*" or "quality improv*" or "Outcome and Process Assess*" or "Quality indicator*" or "quality assess* " or "quality measure* " or "quality control*").ti,ab.	3727733

4	*neurosurgery/ or *head injury/ or *spinal cord injury/ or *traumatic brain injury/	159845

5	("neuro surg*" or "neurosurg*" or "Neurological surg*" or TBI or (("neuro*" or "Brain " or "Cerebro cranial" or "Cerebrocranial" or "cranio cerebral" or "craniocerebral" or "head " or "spinal cord" or "cerebral" or "cerebrovascular") adj1 ("Trauma*" or "Wound*" or "lessi*" or "injur*"))).ti,ab.	399556

6	2 or 3	3866453

7	4 or 5	427785

8	1 and 6 and 7	5534

9	(exp animal/ or nonhuman/) not exp human/	7950931

10	8 not 9	5404

**Web of Science Search Strategy**

\# Database: Web of Science Core Collection

\# Entitlements:

- WOS.SCI: 1945 to 2026

- WOS.AHCI: 1975 to 2026

- WOS.ESCI: 2005 to 2026

- WOS.SSCI: 1956 to 2026

\# Searches:

1: TI=( adolescen* OR child* OR schoolchild* OR infant* OR girl* OR boy OR boys OR boyhood OR teen OR teens OR teenager* OR youth* OR pediatr* OR paediatr* OR puber*)	Results: 2259556

2: TI=("checklist*" or "check list*" or "indicator*" or "framework*" or "tool*" or "score*" or " Evaluate*" or "Screen*" or "Instrument*" or "scale" or "Measure*" or "Protocol" or "survey*" or "questionnair*" or "interview*" or "asses*" or "Guideline*" or "Index" or "safet*")	Results: 5029404

3: TS=(("neuro*" or "Brain " or "Cerebro cranial" or "Cerebrocranial" or "cranio cerebral" or "craniocerebral" or "head " or "spinal cord" or "cerebral" or "cerebrovascular") NEAR ("Trauma*" or "Wound*" or "lessi*" or "injur*"))	Results: 366069

4: TS=("Capacit*" or "Facilit*" or "Resource*" or "Workforce*" or "Personal manag*" or "Staff" or "Trauma center*" or "Treatment approach*" or "Quality Assurance*" or "quality improv*" or "Outcome and Process Assess*" or "Quality indicator*" or "quality assess* " or "quality measure* " or "quality control*")	Results: 6155947

5: #4 AND #3 AND #2	Results: 3685

6: #5 NOT #1	Results: 3293

7: #6 NOT (TI=(veterinary OR rabbit OR rabbits OR animal OR animals OR mouse OR mice OR rodent OR rodents OR rat OR rats OR pig OR pigs OR porcine OR horse* OR equine OR cow OR cows OR bovine OR goat OR goats OR sheep OR ovine OR canine OR dog OR dogs OR feline OR cat OR cats))	Results: 3215
